# Expanding Thermodynamic and Kinetic Frontiers in Molecular Photocatalysis

**DOI:** 10.1021/acscentsci.5c02047

**Published:** 2026-01-07

**Authors:** Dorothee S. Wagner, Leander Spierling, Oliver S. Wenger

**Affiliations:** Department of Chemistry, 27209 University of Basel, St. Johanns-Ring 19, 4056 Basel, Switzerland

## Abstract

Visible photons carry significantly more energy than the thermal energies typically used to overcome activation barriers in conventional chemistry. This thermodynamic advantage enables photochemical reactions that are inaccessible from electronic ground states. However, photochemistry also faces a kinetic challenge: excited states are inherently short-lived, necessitating rapid reactivity before their decay. In this Outlook, we explore the unique interplay of thermodynamics and kinetics in molecular photochemistry. We highlight current limits and knowledge gaps and propose directions for advancing the conceptual framework of photocatalysis. Topics include the design of photocatalysts with extreme redox potentials, the use of solvated electrons and visible-to-UV upconversion, and the potential to bypass Kasha’s rule for higher-energy photochemical processes. Our aim is to survey strategies for pushing the boundaries of photocatalysis and to inspire future conceptual innovation in the field.

## Graphical abstract

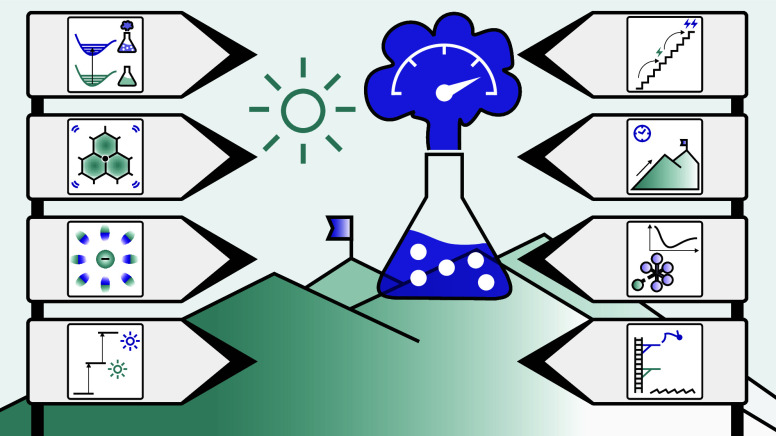

## Introduction

Green photons with a wavelength of 550 nm carry energy equivalent to a thermal temperature of approximately 26,000 K. This comparison highlights the exceptional ability of light to drive chemical reactivity that lies far beyond the capabilities of conventional thermal activation, even though the reaction itself uses only a fraction of the light energy supplied. This fundamental property supports a wide range of transformative applications, including solar fuel generation, photoredox synthesis of fine chemicals and pharmaceuticals,^1^ environmental remediation, and phototherapy in medicine.^2^

While photochemistry offers distinct thermodynamic advantages, it introduces a major kinetic challenge: the conversion of electronic excitation energy into chemical energy must occur before the excitation is lost through non-productive decay.^3,4^ Depending on the nature of the excited states and the chemical environment, this conversion must take place on time scales ranging from picoseconds to milliseconds.^5,6^ As a result, achieving both sufficiently long-lived and at the same time reactive excited states imposes significant constraints on the design of photocatalysts.^7^

While photochemistry offers distinct thermodynamic advantages, it introduces a major kinetic challenge: the conversion of electronic excitation energy into chemical energy must occur before the excitation is lost through non-productive decay.

Another challenge in advancing photochemistry, especially when attempting to push thermodynamic and/or kinetic boundaries, is the identification of the true catalytically active species.^8^ Although this issue is well known in thermally driven catalysis, it becomes even more complex in photochemical systems.^9^ Photoexcited species are often prone to photodegradation under the prolonged irradiation conditions typical in synthetic applications.^10,11^ The resulting degradation products can themselves be photoactive and may contribute to observed reactivity, while eluding straightforward detection.^12^ Although such processes do not necessarily hinder empirical discovery,^13^ they complicate mechanistic analysis and limit the potential for rational catalyst and reaction design.

Modern time-resolved laser spectroscopy provides powerful tools to investigate the initial photoinduced elementary steps of reaction mechanisms.^14,15^ However, the formation of stable photoproducts often involves multiple downstream steps that do not rely on light and are more difficult to probe spectroscopically.^16^ These later stages can be just as important as the initial excitation event in determining the overall reaction outcome.^17,18^ A complete mechanistic understanding requires insight into both photoinduced and thermally driven processes. Encouragingly, recent work has advanced this goal,^5^ with studies elucidating the roles of active photocatalyst identity,^10^ solvated electron formation,^19,20^ photon upconversion,^21^ and mechanistic pathways that challenge Kasha’s rule,^22^ a foundational principle of photochemistry.^23^

In the following section, we provide a conceptual overview of strategies designed to expand the thermodynamic and kinetic scope of photocatalysis. Subsequent sections explore these concepts in greater depth, each concluding with a focused topical outlook. We conclude with a broader perspective on emerging directions and future opportunities in the conceptual development of this field.

## Overview of Different Concepts

In this section, we briefly survey key conceptual approaches aimed at expanding the thermodynamic and kinetic frontiers of photocatalysis. We introduce these strategies through a discussion of Figure 1, proceeding square by square, before examining each concept in greater detail in the sections that follow.

**Figure 1:**
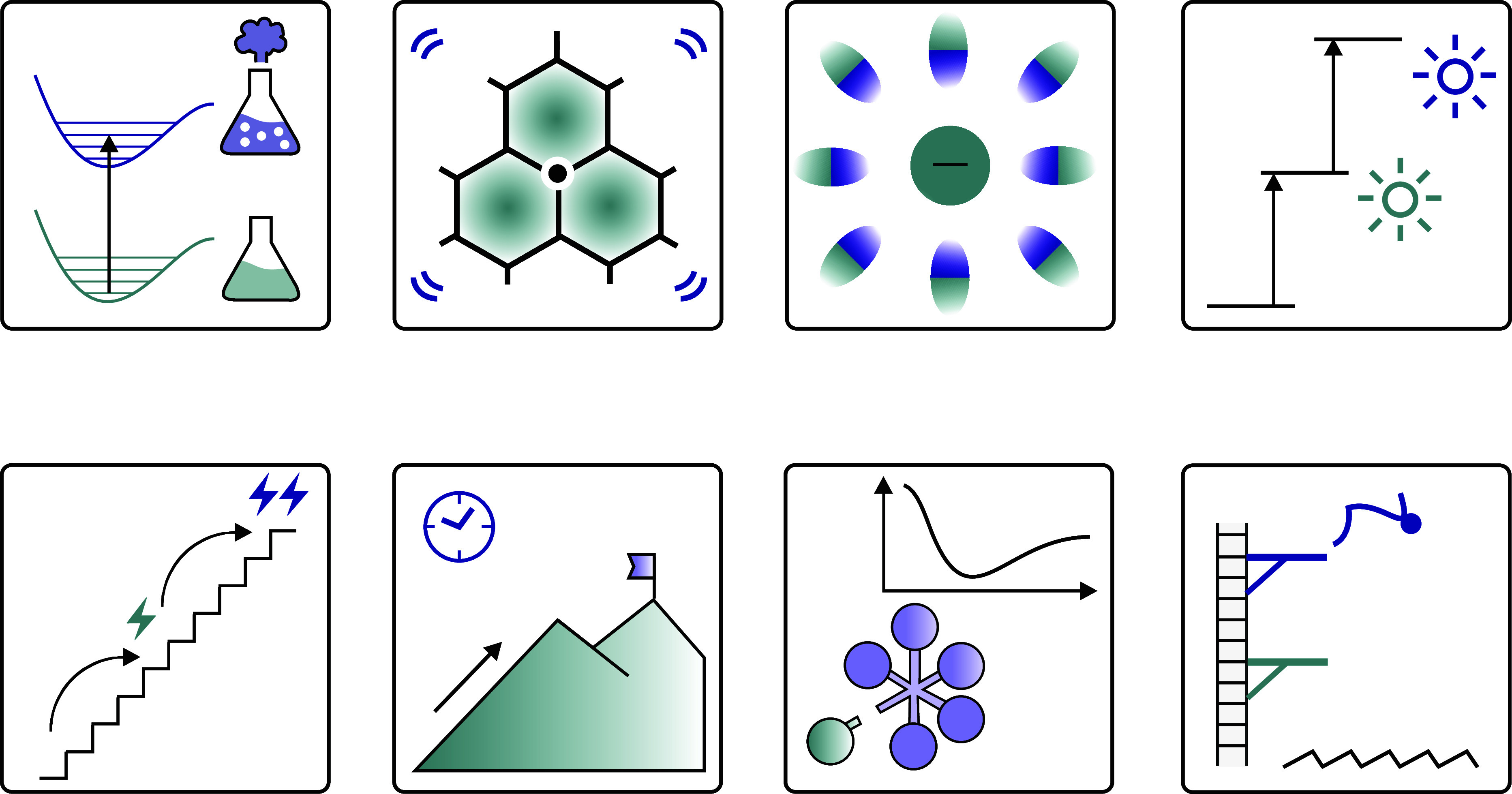
Key concepts and topics discussed in this Outlook: (a) highly reactive excited states in stable photoactive compounds, (b) excited organic radicals, (c) solvated electrons, (d) photon upconversion, (e) redox upconversion, (f) long-lived excited states enabling slow thermodynamically uphill reactions, (g) ultrafast photodissociation reactions, and (h) anti-Kasha reactivity.

Our discussion begins with the design of photocatalysts featuring intrinsically highly reactive excited states (Figure 1a). This line of research has prompted interest in excited organic radicals as potential photocatalysts (Figure 1b) and in the use of solvated electrons in photocatalysis (Figure 1c),^24,25^ whether generated deliberately or arising unintentionally in systems initially thought to operate via excited organic radicals.^26^ From this body of work, multiphoton excitation strategies have also emerged, in which two or more excitation events are required for a single catalytic turnover.^27,28^ Among these, photon upconversion appears particularly promising for enhancing photocatalytic performance (Figure 1d).^21^ More recently, the concept of redox upconversion has been introduced as a strategy for driving thermodynamically demanding reactions.^29^ In this approach, a redox species with greater oxidizing or reducing power than the initial reagent is generated during the course of the reaction (Figure 1e).

We share our current perspective on how mechanistic photocatalysis may further develop and what conceptual directions could be anticipated, with the goal of contributing to a holistic approach to the advancement of photochemistry.

The kinetic counterpart to developing more reactive excited states is the design of photocatalysts with exceptionally long excited-state lifetimes. These extended lifetimes enable processes that remain thermodynamically uphill even after photoexcitation (Figure 1f).^30^ At the opposite extreme, photodissociation reactions can proceed from ultrashort-lived excited states, most notably from ligand-to-metal charge transfer (LMCT) states in transition metal complexes (Figure 1g).^15,31^ These light-induced dissociation events offer a promising route to directly channel excitation energy into productive chemical transformations. Finally, recent studies have begun to challenge Kasha’s rule,^22^ which implies that photochemical reactivity generally occurs only from the lowest excited state of a given spin multiplicity.^32^ While such anti-Kasha reactivity often arises at the kinetic limit for bimolecular reactions (Figure 1h), these exceptions offer intriguing opportunities to surpass conventional thermodynamic constraints.

## Photocatalysts Featuring Highly Reactive Excited States

In this context, the term “reactive” refers to species with exceptionally strong reducing or oxidizing capabilities, or with high excited-state energies (Figure 2b), ideally coupled with lifetimes sufficient to support diffusion-controlled bimolecular reactions. Systems operating via ordinary monophotonic excitation are considered in this section.

**Figure 2:**
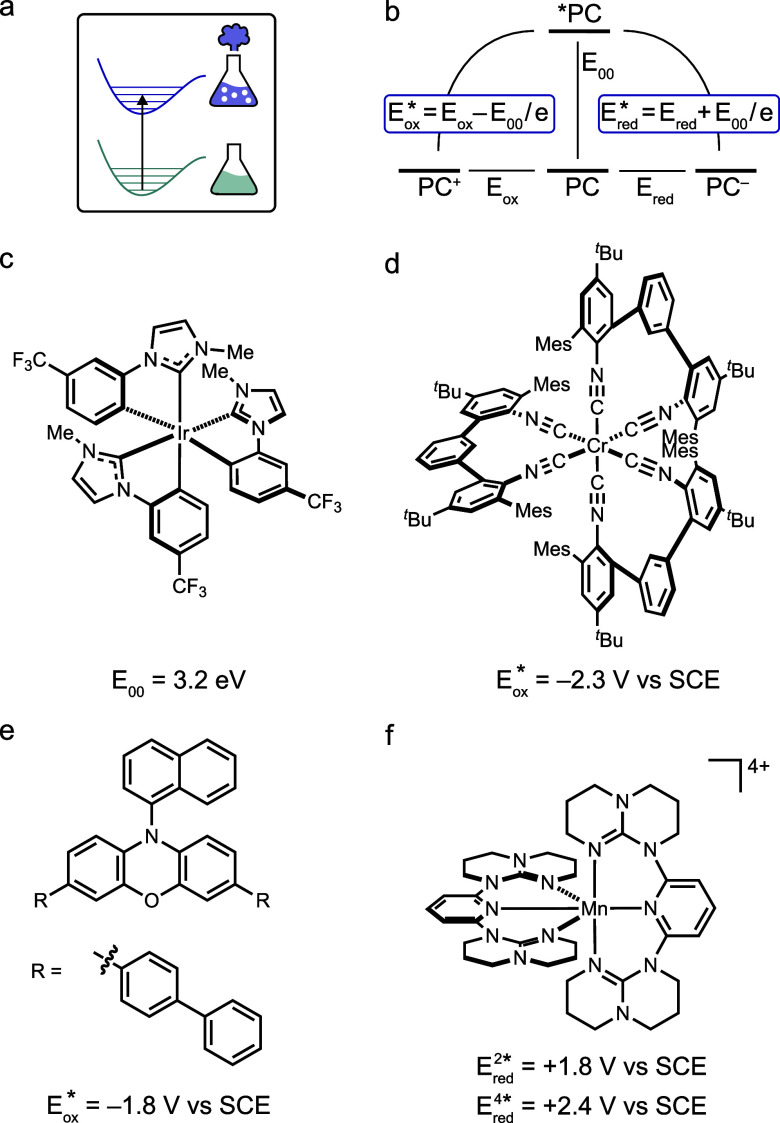
(a) Pictogram from Figure 1a. (b) Latimer diagram with ground and excited state properties of a photocatalyst (PC). Molecular photocatalysts featuring highly reactive excited states: (c) an Ir^III^ complex with an exceptionally high triplet excited-state energy,^33,34^ (d) a Cr^0^ super-reductant operating under red light,^35^ (e) an organic super-reductant,^36^ and (f) a Mn^IV^ superoxidant.^37^

Many well-known organic chromophores possess high-energy singlet excited states that facilitate photoreactivity.^3^ More challenging, however, is the design of chromophores with high-energy triplet excited states, which can enable transformations that are inaccessible from the singlet manifold.^38^ Recent interest in triplet–triplet energy transfer catalysis has spurred the development of photocatalysts featuring high-energy triplet states, in some cases exceeding 3.3 eV (Figure 2c), corresponding to the energy of ultraviolet photons.^33,34^ To date, research in this area has primarily focused on Ir^III^ complexes and a handful of long-established organic chromophores, but significant opportunities for innovation remain.^33,34,39,40^ Although ultraviolet-light-driven photochemistry has historically been viewed as unselective and synthetically impractical, recent advances demonstrate its potential for enabling otherwise inaccessible chemical transformations.^41^

Electron-rich metal complexes and organic compounds can act as powerful photoreductants upon electronic excitation (Figure 2d,e).^35,42^ This concept has been extensively explored over the past decade, with dehalogenation reactions commonly serving as benchmark transformations.^43,44^ The greater a compound’s ground-state reducing power, the lower its excited-state energy can be, and thus the lower the photon energy required to achieve potent photoreducing behavior, and *vice versa*. Under red-light irradiation, the Cr^0^ complex shown in Figure 2d promotes reductive dehalogenations that typically require blue or UV light when using other photocatalysts.^35^ In such cases, regenerating the catalyst’s initial oxidation state demands a stronger terminal reductant. While this might seem disadvantageous, it can be compatible with overall redox-neutral processes.^45,46^ In some instances, a radical chain mechanism may further enhance reactivity.^47^ In particularly extreme examples, the excited photoreductant can reduce the solvent or even generate solvated electrons (see below).

While the limits of photoreductive behavior have been extensively explored using a wide range of photocatalysts,^48^ systems where a substrate is oxidized remain less thoroughly studied. Often, sacrificial electron donors (with no synthetically increased value) are oxidized to transform the excited photocatalyst to a potent ground state reduction catalyst. Thus, for substrate oxidation there is still considerable room for innovation. Complexes of electron-deficient transition metals are especially promising. For example, a Mn^IV^ complex is capable of oxidizing acetonitrile (Figure 2f), and Re^II^, Ce^IV^ and W^VI^ compounds have also shown strong oxidizing ability.^49–51^ These systems offer stable, well-defined superoxidants that complement organic radical cation-based oxidants (see below), whose often ambiguous mechanisms can present a disadvantage in terms of mechanistic understanding and control.^52^

## Excited Organic Radicals as Potential Photocatalysts

Photoinduced electron transfer (PET) reactions from excited organic radicals attracted attention from the physical chemistry community well before their rise in modern synthetic photochemistry.^53–55^ More recently, interest in excited organic radicals has grown with the development of the so-called consecutive photoinduced electron transfer (ConPET) mechanism.^56^ In this process, an initial PET step generates an organic radical, which then undergoes a second PET event with a substrate molecule using the accumulated energy of two photons to achieve extremely high redox potentials (Figure 3b).

**Figure 3:**
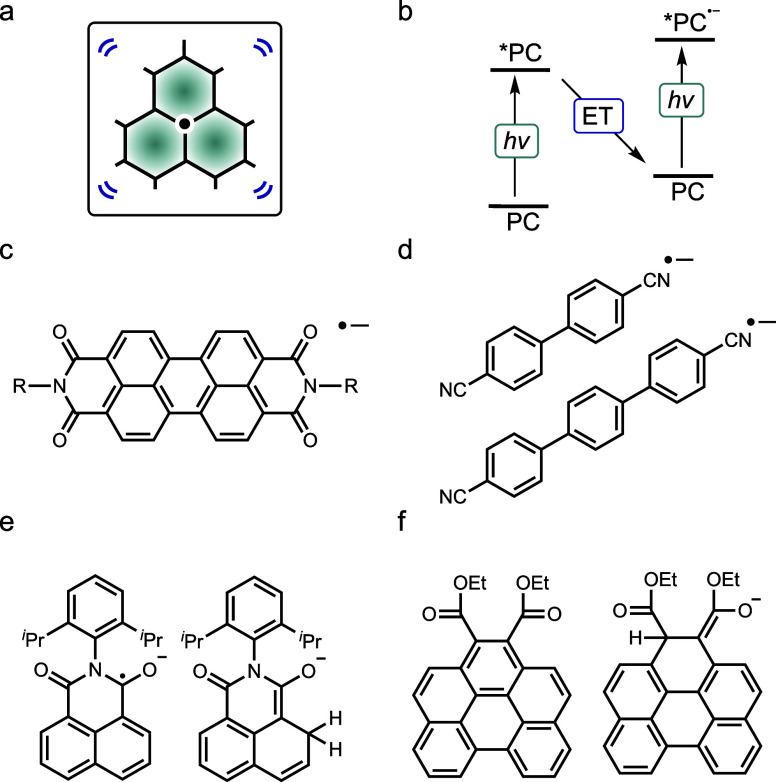
(a) Pictogram from Figure 1b. (b) Simplified ConPET energy diagram (ET = electron transfer), a process often used to generate excited radicals. (c) The perylene diimide (PDI) radical anion, initially proposed as the key photoactive species in ConPET,^56^ later re-evaluated through mechanistic studies.^12,57,58^ (d) Dicyanoarene radical anions, for which direct photoreaction from the excited radical state to substrate molecules has been observed via transient absorption spectroscopy.^59^ (e) Benzo­[ghi]­perylene monoimide precatalyst and its ring-opened photoproduct, which appears to function as the true photocatalyst.^60^ (f) A related monoimide radical anion precursor and one of its photoactive closed-shell decomposition products.^10,61^

After seminal mechanistically oriented work,^54,55,62,63^ an early synthetically focused study hypothesized to use perylene diimide radical anions (Figure 3c) as key intermediates,^56^ sparking further research into rylene radical anions.^57,61,62,64,65^ This included their generation through electrochemical methods, often referred to as electron-primed photoredox catalysis or electrophotocatalysis.^66^ Subsequent investigations expanded to other radical systems,^67^ such as acridinyl and boryl radicals,^13,68^ rhodamine derivatives,^69^ and radicals derived from other organic dyes.^70,71^

Mechanistic studies across these systems have revealed a level of complexity that surpasses the assumptions made in early synthetic work. While initial proposals suggested that excited organic radicals directly engage in PET with substrate molecules, this has been experimentally confirmed only in a few cases such as for the aryl radicals in Figure 3d.^52,57–59,72^ A growing body of evidence indicates that many of these radicals degrade into closed-shell species (two-electron reduced, singly protonated) or charge-transfer complexes (Figure 3e,f),^10,12,24,60,73–77^ which possess significantly longer-lived excited states and are likely responsible for much of the observed photochemistry. Furthermore, the formation of solvated electrons (see below) is increasingly recognized as a viable additional pathway in these systems. Overall, the idea that such reactions proceed through a single, uniform mechanism appears to be overly simplistic. Multiple pathways may operate in parallel, with their relative contributions evolving over the course of the reaction.^59,78^

Photoreactions of organic radical cations are less thoroughly studied than those of radical anions and, in most cases, rely on their electrochemical generation.^79,80^ ConPET processes involving radical cations remain underdeveloped,^52,81–83^ and mechanistic investigations are significantly fewer compared to those focused on radical anions.^52,84^ Further research into radical cations may therefore be warranted in the future.^66^

## Solvated Electrons as Key Drivers of Photochemical Reactions

Solvated electrons were initially generated using ultraviolet and higher-energy radiation, but more recently have become accessible through multiphoton excitation pathways.^85^ In these approaches, classical Ru^II^ and Ir^III^ complexes are irradiated with visible light, leading to their photoionization during a second excitation step, following the formation of a long-lived excited state or a persistent photoreduction product (Figure 4b).^26,86^ For the Ir^III^ complex in Figure 4c this represents an example of a higher energy state photoreactivity (anti-Kasha) similar to what will be discussed later in the text. In deaerated, alkaline water, the hydrated electron has a reduction potential of −2.9 V versus NHE and a lifetime of approximately 1.5 μs. Their formation and reactivity have been studied using transient UV–visible absorption spectroscopy.^87,88^

**Figure 4:**
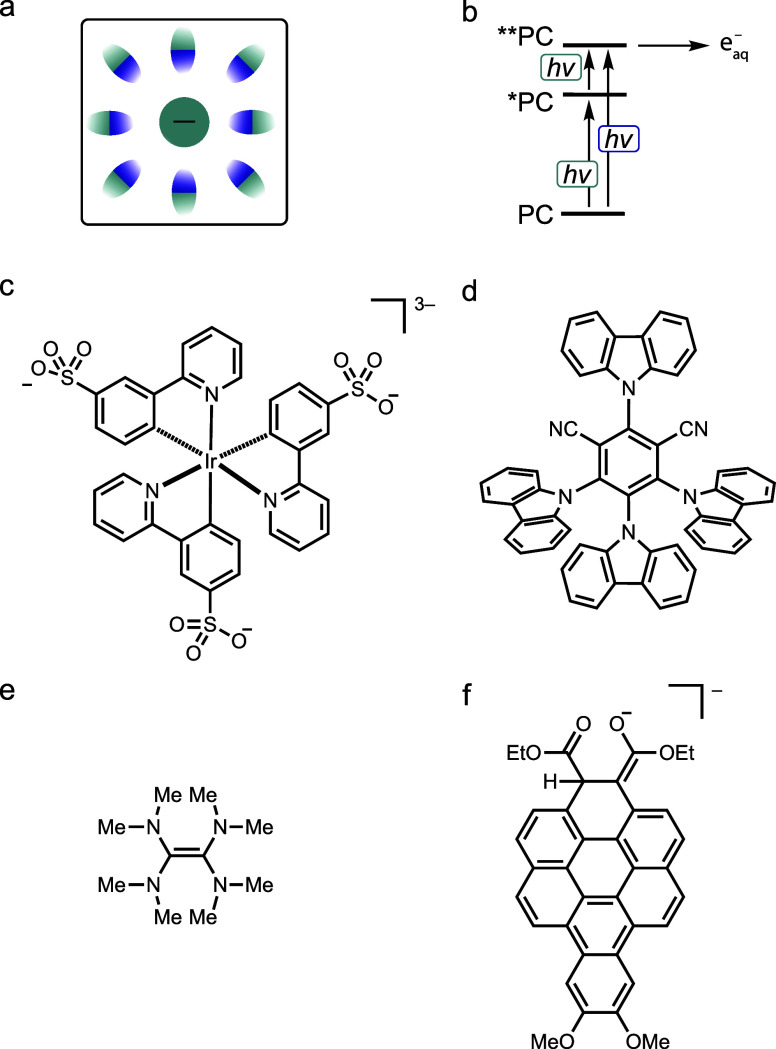
(a) Pictogram from Figure 1c. (b) Simplified energy scheme for higher state excitation (**PC) followed by ionization and solvated electron formation (e^–^_aq_). (c) Ir^III^ complex enabling the generation of hydrated electrons.^26^ (d) Donor–acceptor cyanoarene facilitating the formation of solvated electrons in acetonitrile.^20^ (e) Tetrakis­(dimethylamino)­ethylene (TDAE) leading to acetone solvent reduction upon direct excitation.^90^ (f) Coronene enolate, a two-electron, one-proton reduction product accessible via visible-light excitation of a diester precursor, capable of reducing benzene.^73^

More recently, solvated electrons have been identified as key catalytic species in synthetic applications, such as Birch-type reductions carried out under mild conditions.^77^ Solvated electrons may be more common than previously thought,^78,89^ although their direct detection in organic solvents is more challenging than in water. This has been illustrated by recent studies involving donor–acceptor cyanoarenes (Figure 4d).^20^

Solvated electrons exist only in select solvents (such as water or acetonitrile), whereas other solvents are simply reduced themselves. In such cases, the reduction potential of the solvent becomes the limiting factor for the achievable reducing power, similar to how solvent properties constrain Brønsted acidity. A relevant example is the formation of ketyl radicals in acetone following direct excitation of the commercial superphotoreductant TDAE (Figure 4e).^90^ A particularly compelling case involves a benzo­[a]­coronene enolate (Figure 4f), which becomes photochemically accessible upon excitation of a diester precursor and can reduce benzene.^73^ This system enables Birch-type reductions with improved efficiency and broader substrate scope compared to earlier methods. The photoactive catalytic species in this case resembles the type of degradation product that can form when using the ConPET strategy under reductive conditions.^10^

Conceptually related photooxidizing reactivity involving the solvent has also been observed, although less frequently than photoreductions. An example is provided by a Mn^IV^ complex (Figure 2f), which can oxidize acetonitrile upon photoexcitation.^37^

The field would benefit from a clearer understanding of the reactions in which solvated electrons play a key role in driving photochemistry, including their direct spectroscopic detection, as well as the development of further photoactive compounds capable of generating solvated electrons under visible light with high quantum yields.

## Photon Upconversion to Drive Ultraviolet-Dependent Reactions with Visible Light

The term *photon upconversion* encompasses several distinct mechanistic pathways.^91^ In photochemical contexts, triplet excited states are often generated on organic molecules through energy transfer sensitization by a suitable sensitizer.^92^ These triplet states can undergo *triplet–triplet annihilation* (TTA), a process in which one of two annihilators is promoted to its lowest singlet excited state, possessing up to twice the energy of the triplet state (Figure 5b). The fundamental principles underlying this mechanism have been extensively reviewed in the literature.^21,93–96^

**Figure 5:**
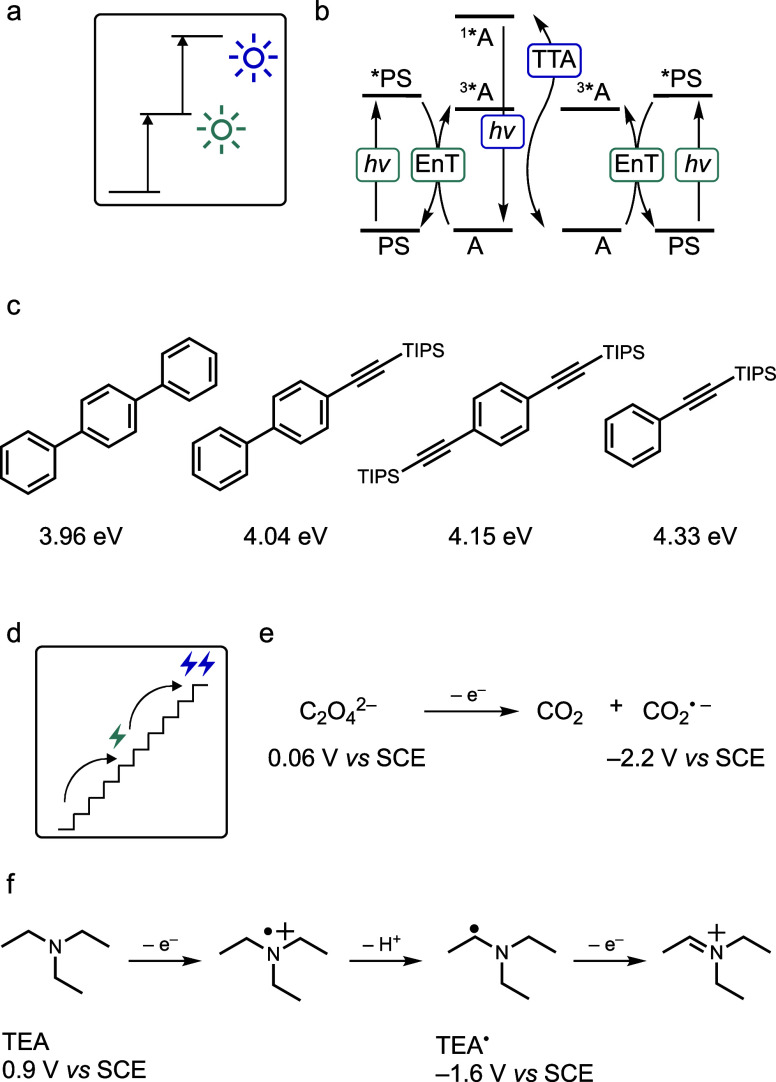
(a) Pictogram from Figure 1d. (b) Simplified sensitized triplet triplet annihilation upconversion (sTTA-UC) scheme. PS = photosensitizer, A = annihilator, EnT = energy transfer (c) Annihilator molecules used recently for upconversion to the UV, along with the corresponding upconverted energies achieved (TIPS = triisopropylsilyl).^103,104,107^ (d) Pictogram from Figure 1e. (e) The concept of redox upconversion illustrated by the reducing powers of the oxalate dianion and (f) triethylamine (TEA) and their one-electron oxidation products, the carbon dioxide radical anion and the α-amino alkyl radical TEA^•^.^110,111^

TTA upconversion has emerged as a key mechanism underlying several biphotonic reactions and has consequently attracted increasing interest in synthetic studies aimed at harnessing low-energy red light to drive photoreactions that normally require higher-energy green or blue excitation.^97^ Significant attention has been devoted to photoreductions,^98^ while photooxidations have seen comparatively less innovation.^99,100^ Photon upconversion holds considerable promise for enabling ultraviolet-dependent photochemistry.^21^ Although UV excitation remains the most straightforward method for such reactions,^41^ there are situations where UV light may be absorbed by unintended reaction components or where direct UV excitation causes too much photodamage.^21,101,102^

Recent work has achieved upconversion from the UV-A region (355/375 nm) to the UV-C range, setting a new record for upconverted energy at 4.33 eV (Figure 5c).^103,104^ This upconverted light can, in principle, be used directly for UV photochemistry involving singlet excited states, such as photochemical permutation reactions.^105^ However, accessing the substrates’ higher excited triplet states following upconversion remains more challenging. To date, proof-of-principle studies have demonstrated photocleavage reactions and intramolecular Paternò–Büchi reactions using this approach,^106,107^ but there is significant room for innovation in more synthetically focused applications of upconversion. Despite relying on relatively low-energy inputs and non-coherent light, the overall efficiency of the upconversion process could limit its viability in real-world applications.^27,108,109^ A key challenge moving forward is thus to enhance the quantum yields of visible-to-UV upconversion processes.

## Redox Upconversion

Many photoredox reactions proceed with remarkable efficiency despite thermodynamically unfavorable electron transfer elementary steps. This can be attributed to redox upconversion,^29^ a recently introduced concept describing the conversion of weaker reductants or oxidants into stronger ones in the catalytic cycle.^112^ The core idea of redox upconversion is to couple an energetically uphill transformation that generates a highly reactive species with an exergonic process that yields a stable coproduct, a principle well established in biochemistry.^113^

For example, one-electron oxidation of electron donors such as oxalate or triethylamine can produce species like CO_2_^•–^ radical anions (Figure 5e)^110^ or α-amino alkyl radicals (Figure 5f), formed after deprotonation of the oxidized amine, which are significantly more reducing than their parent compounds themselves.^114,115^ The driving force for these reactions comes from the formation of CO_2_ and iminium cations, respectively. When carefully designed, such systems can create exceptional redox power for light-independent chemical steps following photochemical initiation. Reactions of this type often exhibit overall quantum yields far greater than 1, sometimes reaching values around 100, due to radical chain propagation induced by the redox upconverted species.^5,47^ Although significant progress has been made in this area,^116^ there is still considerable potential for further innovation, particularly in accessing extreme redox potentials of ground-state species under mild reaction conditions.

Further challenges include designing efficient radical cascades to enhance the overall quantum yields of photoredox reactions and to offset elementary steps with inherently low quantum yields.^117^ For example, the loss of CO_2_ from carboxyl radicals generated via low quantum yield metal–ligand bond photocleavage upon ligand-to-metal charge-transfer (LMCT) excitation of electron-deficient metal complexes,^118,119^ a topic of current interest (see below), can produce upconverted radicals whose reactivity may compensate for the inefficiency of the initial photochemical step. LMCT photocatalysis is gaining traction in synthetic chemistry, and these strategies have already attracted attention while offering promising potential for further development.^120,121^

## Uphill Reactions Enabled by Unusually Long-Lived Excited States

The Rehm–Weller experiment, conducted over 50 years ago, showed that the rates of bimolecular electron transfer reactions depend strongly on the free energy change of the process, often referred to as the driving force.^122^ According to the Rehm–Weller behavior, many photoredox reactions rely on an initial PET step with a driving force of 0.2 eV or more, where electron transfer occurs at or near the diffusion limit.^122^ Under these conditions, even photocatalysts with excited-state lifetimes in the low nanosecond range can efficiently promote photoredox chemistry.

Most photosensitizers have lifetimes ranging from a few nanoseconds to the low microsecond range,^4,123^ enabling photoinduced electron transfer reactions with lower driving forces. A recent study using a Cr^III^ photocatalyst (Figure 6c) with an excited-state lifetime of 23 μs demonstrated photoreductions proceeding through an initial photoinduced electron transfer step that was 0.5 eV uphill.^30^

**Figure 6:**
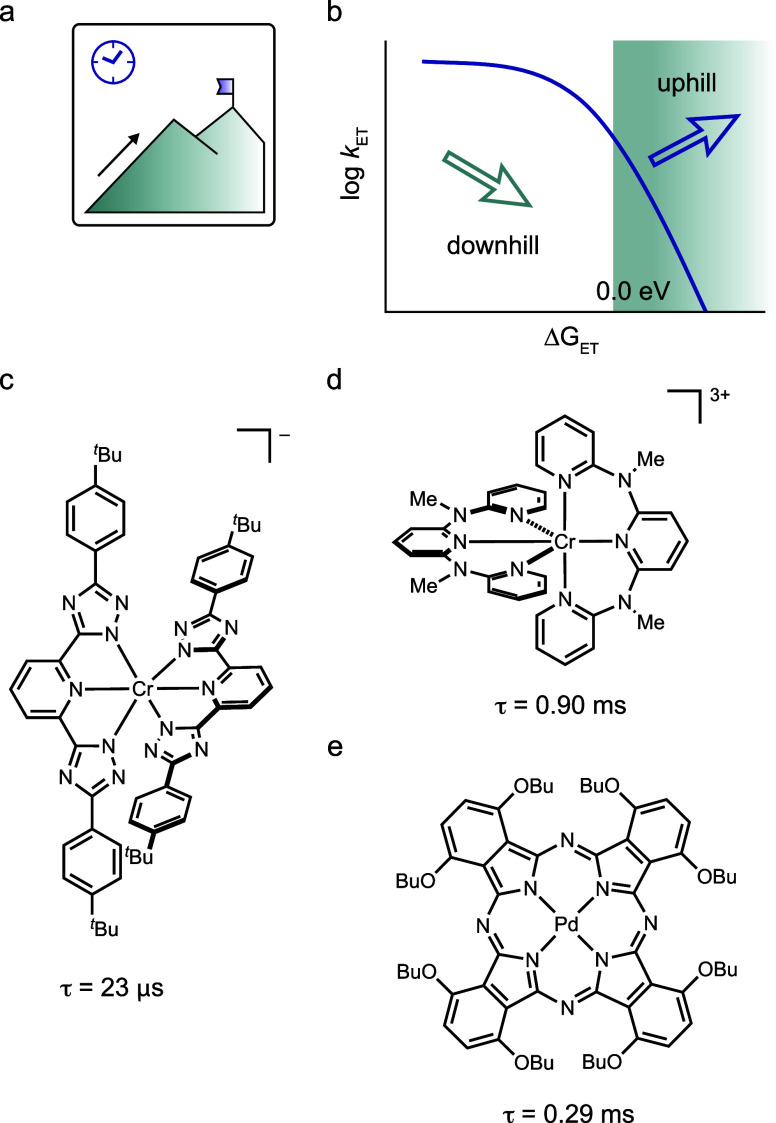
(a) Pictogram from Figure 1f. (b) Rehm–Weller plot of the ET rate vs the driving force. (c)–(e) Photocatalysts with long excited-state lifetimes enabling endergonic photoinduced electron transfer: (c) Cr^III^ complex for up to 0.5 eV uphill reactions,^30^ (d) Cr^III^ complex with longer lifetime potentially allowing more endergonic reactions,^124^ and (e) representative Pd phthalocyanine compound.^125^

This behavior can be explained by the Rehm–Weller experiment, where the quenching rate is lowered for uphill reactions but does not drop to zero immediately. Therefore, even with low quenching constants for uphill reactions, long-lived excited states can show quenching efficiencies suitable for photochemical reactions (see Figure 6b). In another recent study, an uphill reaction between a cationic Ir^III^ photocatalyst and the BAr_4_F^–^ anion has been investigated, where the uphill reactivity has been attributed to electrostatic interaction and the resulting Born and Coulomb correction terms to the Gibbs free energy.^126^

Other Cr^III^ complexes (Figure 6d),^124^ phthalocyanine derivatives (Figure 6e),^125,127^ and selected organic photocatalysts exhibit excited-state lifetimes up to the millisecond range,^128^ which could in principle enable even more strongly endergonic bimolecular electron transfer reactions.

A similar line of reasoning applies to reactions initiated by triplet–triplet energy transfer. The observation of visible-light-mediated dearomative cycloaddition reactions of heterocycles that are uphill by 0.3 eV from the used Ir^III^ photosensitizer can therefore be explained by the Boltzmann behavior as described in the Rehm–Weller equation.^129^ This suggests that the basic principles discussed in this section may be less widely recognized than one might assume, indicating potential for synthetic innovation based on a clearer understanding of this concept.

To that effect, deviations from Rehm–Weller behavior have recently been observed (with an unexpected *lack* of reactivity),^130,131^ refining the mechanistic understanding of the theory, yet these do not represent uphill reactions and thus differ from the theory–consistent examples we focus on here.

Uphill photoinduced elementary steps may also benefit from reduced in-cage charge recombination, a process in which geminate radical pairs formed after electron transfer undergo back electron transfer within the solvent cage before they can separate. While it remains under investigation to what extent cage escape quantum yields depend on the driving force,^132^ it is plausible that uphill PET could enhance cage escape. In such cases, charge recombination may fall into the Marcus inverted regime, where reverse electron transfer becomes slower than escape from the solvent cage.^133,134^ This interplay between productive but slow endergonic electron transfer and energy-wasting charge recombination may be broadly relevant for improving photoredox catalysis.^30,114^

## Photochemistry in the Primary Coordination Sphere of Metal Complexes

Electronic excitation in coordination complexes redistributes electron density between the metal and its ligands, often significantly altering the strength of metal–ligand bonds. Metal-centered excitations into antibonding orbitals can lead to the release of small neutral molecules relevant for phototherapy,^135^ while ligand-to-metal charge transfer (LMCT) excitations often produce organic radicals through ligand release, enabling further reactivity (Figure 7b).^31,118^ LMCT processes underpin the classical ferrioxalate actinometer and have recently attracted interest as a foundation for LMCT catalysis in synthetic chemistry.^31,121,136^ This typically involves radical pathways on the ground-state potential energy surface, initiated by photochemical metal–ligand bond cleavage, a step that is often neglected and may be inefficient.

**Figure 7:**
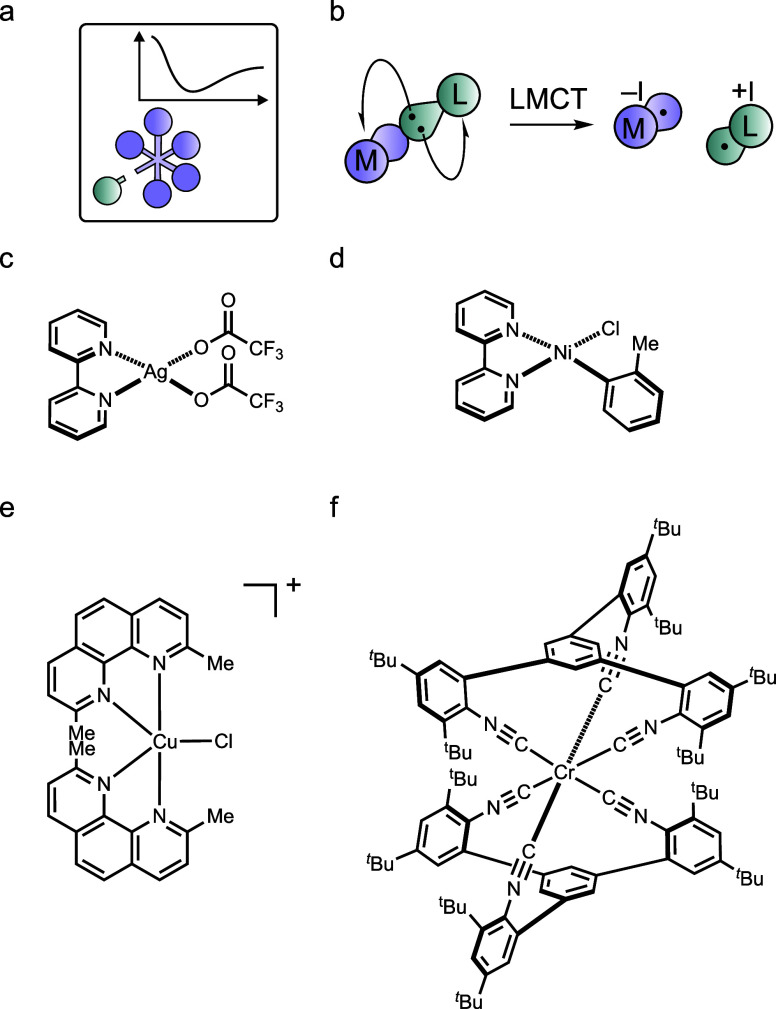
(a) Pictogram from Figure 1g. (b) Visualization of LMCT bond breaking. (c)–(f) Selected metal complexes in which the photochemical elementary step underlying LMCT catalysis has been investigated: (c) [Ag­(bpy)­(CF_3_COO)_2_],^137^ (d) [Ni­(bpy)­(*o*-tolyl)­Cl)],^138^ (e) [Cu­(dmp)_2_Cl]^+^.^139^ and (f) Cr^0^ complex undergoing reversible photodissociation of one arylisocyanide coordination unit from an MLCT excited state.^140^

Complexes of metals in high oxidation states such as W^VI^, Ce^IV^, Fe^III^, and Cu^II^, coordinated by carboxylates, other oxygen or nitrogen donors, or halides, have been studied extensively in this context.^141–143^ However, detailed insights into the initial photochemical events remain limited (Figure 7c–e).^137–139,144,145^ Although foundational work on photodissociation in metal complexes exists,^135,146^ modern ultrafast methods such as transient infrared and ultraviolet–visible spectroscopy now allow more direct investigation.^15,31^ Compounds that undergo reversible photoinduced ligand dissociation are especially attractive for this purpose. Although there are well-studied systems such as in the reversible cleavage of a Ni–C bond or a Cr–C bond, these systems remain rare. (Figure 7f).^140,147^

In several systems, the quantum yield of photocleavage strongly depends on excitation wavelength, suggesting that bond dissociation may not originate from the lowest excited state of a given spin multiplicity.^138^ This points to a violation of Kasha’s rule. While such behavior is more intuitive for dissociative reactions than for bimolecular processes,^148^ the mechanisms remain poorly understood and warrant further study.

## Breaking Kasha’s Rule

Kasha’s rule was originally formulated to describe the observation that luminescence typically occurs only from the lowest electronically excited state of a given spin multiplicity.^23^ Although it has been argued that Kasha’s original definition should remain unchanged,^149^ photochemists recognized early on that both luminescence and photochemical reactions rely on electronically excited states with sufficiently long lifetimes.^32^ Consequently, much of the modern research literature applies the term anti-Kasha behavior also to the broader field of photochemistry. It is important to distinguish true exceptions to Kasha’s rule from artifacts and other trivial causes of apparent deviations,^148^ as not every excitation into higher energy absorption bands constitutes a breaking of Kasha’s rule.

Anti-Kasha behavior has been proposed in several cases of photoredox catalysis to explain the formation of products that would be unexpected from the lowest electronically excited state,^84^ but until recently there had been no direct spectroscopic evidence for it. In a recent study, higher excited state reactivity of a terphenyl radical anion (Figure 3d) was directly observed by transient UV–visible absorption spectroscopy, while excitation into the lowest excited state led to markedly weaker reactivity or no reaction at all, depending on the substrate.^22,59^ Pre-association between the terphenyl photocatalyst and the substrate molecules, as well as careful tuning of the driving force for photoinduced electron transfer and its reorganization energy, turned out to be essential to bypass Kasha’s rule and to enable chemical reactivity before energy dissipation occurred.^22,59^ Much remains to be understood about this complex interplay between thermodynamics and kinetics on higher excited state potential energy surfaces.

The terphenyl anion case exemplifies one of the potential key advantages of anti-Kasha behavior, namely that nearly twice the energy can be made available from a higher excited state than from the lowest excited state. In other examples where anti-dissipative strategies have been explored, the achievable energy gains were substantially smaller,^150–153^ yet the concept remains highly intriguing and worthy of further investigation from both experimental and computational perspectives.^154,155^

## Conclusions and Outlook

Considering the eight different concepts and topics from Figure 1, we identify the following research directions that warrant further investigation in future studies:

- In the development of reactive excited states in metal complexes and closed-shell organic compounds, research has so far tended to focus more on photoreductions than on photooxidations.^43^ This suggests that there may be greater potential for innovation in the design of highly oxidizing excited states compared to strongly reducing ones.
- The photoreactivity of organic radicals is considerably more complex than initially assumed in synthetic studies that began emerging 11 years ago. Multiple mechanistic pathways can operate simultaneously, and their relative importance may shift over time.^22,59^ To achieve genuine photoreactivity from excited organic radicals, developing pre-aggregation strategies between the photoactive species and substrate molecules to facilitate the required picosecond-scale reactivity may represent a promising direction for future research.^24^
- Solvated electrons appear to play crucial roles in photoredox chemistry more often than previously anticipated. A systematic approach to their direct and unambiguous detection would help clarify reaction mechanisms and support more informed photochemical reaction design. While photoactive compounds capable of generating solvated electrons with visible light have already been developed, additional systems achieving higher quantum yields are still needed.
- Photon upconversion may continue to be valuable in scenarios where low-energy red or near-infrared light is particularly advantageous. However, the greatest potential for innovation likely lies in enabling access to the UV range using visible light. Although thermodynamic limits have been impressively extended,^21^ developing visible-to-UV upconversion systems with high quantum yields remains a major goal. Another key challenge is finding more effective ways to access higher excited state triplet photochemistry following upconversion,^106,107^ which typically produces singlet excited states.
- Redox upconversion can enhance photon-to-electron conversion in photocatalysis and may be particularly effective when coupled with photochemical elementary reaction steps that proceed with low quantum yields. The most conceptually intriguing redox-upconverted steps are often “dark reactions” that do not require further light input. Potential for innovation may lie in the identification of systems featuring redox upconversion with a higher potential difference. Another research direction could focus on developing oxidative redox upconversion systems to complement the many reductive examples studied already.
- Reactions from excited states that proceed uphill by 0.5 eV or slightly more are readily achievable, provided the excited states are sufficiently long-lived to show reasonable quenching efficiencies following the Rehm–Weller behavior.^30,129^ The interplay between slow, endergonic but productive photoinduced electron transfer and strongly exergonic, but Marcus-inverted and therefore slow, thermal reverse electron transfer within the solvent cage remains poorly understood. There is likely a trade-off between efficient quenching and inefficient cage escape, which will ultimately influence overall photoredox quantum yields.^133^
- Modern ligand-to-metal charge transfer (LMCT) catalysis, as applied in synthetic contexts, often appears to rely on cleverly designed ground-state radical chemistry, while the actual photochemical elementary steps receive less attention. A substantial body of earlier literature exists on photodissociation reactions in transition metal complexes, and revisiting these fundamental processes with modern time-resolved methods could prove highly valuable.^15^ Quantum yields are expected to depend strongly on excitation wavelength and may reveal unanticipated reactivity arising from higher excited states.
- Kasha’s rule remains one of the most significant conceptual frontiers in photochemistry. It is almost invariably the starting point for photochemical reaction design, as typically only the lowest excited state of a given spin multiplicity is sufficiently long-lived to support bimolecular photochemical processes. While known exceptions to Kasha’s rule have largely been limited to unimolecular reactions and to higher excited state luminescence, recent studies have shown that rational design of bimolecular anti-Kasha photoreactivity is indeed feasible.^22^ A central challenge lies in understanding the elementary aspects of photocatalyst and substrate pre-association,^59^ which allows for substantial mutual electronic coupling, as well as the interplay between driving force and reorganization energy in photoinduced electron transfer from higher excited states.

Continued exchange between synthetic and mechanistically oriented photochemistry remains essential for advancing the field as a whole.

Photochemistry has made remarkable progress over the past decade. From our perspective, this is largely due to the convergence of multiple subdisciplines within chemistry, including synthetic organic chemistry, physical inorganic chemistry, analytical chemistry, and polymer chemistry, all of which have developed a shared interest in the field. Photochemistry also offers promising solutions to current scientific, economic, societal, and environmental challenges. Viewpoints and research priorities often differ significantly between synthetic organic and physical inorganic chemistry and have led to several intensely debated and sometimes controversial discussions.^8,9,28,156–161^ Therefore, continued exchange between synthetic and mechanistically oriented photochemistry remains essential for advancing the field as a whole.

Synthetic innovation is critically important and appears to be advancing more rapidly than mechanistic understanding and conceptual development. With this Outlook, we share our current perspective on how mechanistic photocatalysis may further develop and what conceptual directions could be anticipated, with the goal of contributing to a holistic approach to the advancement of photochemistry.^162^
